# 6′-O-Galloylpaeoniflorin Attenuates Osteoclasto-genesis and Relieves Ovariectomy-Induced Osteoporosis by Inhibiting Reactive Oxygen Species and MAPKs/c-Fos/NFATc1 Signaling Pathway

**DOI:** 10.3389/fphar.2021.641277

**Published:** 2021-04-07

**Authors:** Wenjie Liu, Gang Xie, Guixin Yuan, Dantao Xie, Zhen Lian, Zihong Lin, Jiajie Ye, Wenyun Zhou, Weijun Zhou, Henghui Li, Xinjia Wang, Haotian Feng, Ying Liu, Guanfeng Yao

**Affiliations:** ^1^ Department of Orthopedics, The Second Affiliated Hospital, Shantou University Medical College, Shantou, China; ^2^ Institute of Translational Medicine, Shanghai University, Shanghai, China; ^3^ Guangxi Key Laboratory of Regenerative Medicine, Guangxi Medical University, Nanning, China; ^4^ Guangxi Collaborative Innovation Center for Biomedicine, Guangxi Medical University, Nanning, China; ^5^ School of Biomedical Sciences, The University of Western Australia, Perth, WA, Australia

**Keywords:** 6′-O-galloylpaeoniflorin, osteoporosis, osteoclast, MAPKs, NFATc1, reactive oxygen species

## Abstract

Emerging evidence suggests bright prospects of some natural antioxidants in the treatment of osteoporosis. 6′-O-Galloylpaeoniflorin (GPF), an antioxidant isolated from peony roots (one of very widely used Oriental medicines, with various anti-inflammatory, antitumor, and antioxidant activities), shows a series of potential clinical applications. However, its effects on osteoporosis remain poorly investigated. The current study aimed to explore whether GPF can attenuate osteoclastogenesis and relieve ovariectomy-induced osteoporosis via attenuating reactive oxygen species (ROS), and investigate the possible mechanism. After the culture of primary murine bone marrow-derived macrophages/monocytes were induced by the use of macrophage colony-stimulating factor (M-CSF) and the receptor activator of NF-κB ligand (RANKL) and then treated with GPF. Cell proliferation and viability were assessed by Cell Counting Kit-8 (CCK-8) assay. Thereafter, the role of GPF in the production of osteoclasts and the osteogenic resorption of mature osteoclasts were evaluated by tartrate-resistant acid phosphatase (TRAP) staining, podosome belt formation, and resorption pit assay. Western blotting and qRT-PCR examination were performed to evaluate proteins’ generation and osteoclast-specific gene levels, respectively. The ROS generation in cells was measured *in vitro* by 2′,7′-Dichlorodi-hydrofluorescein diacetate (DCFH-DA). Ovariectomy-induced osteoporosis mouse administered with GPF or vehicle was performed to explore the *in vivo* potential of GPF, then a micro-CT scan was performed in combination with histological examination for further analysis. GPF suppressed the formation of osteoclasts and podosome belts, as well as bone resorption when induced by RANKL through affecting intracellular ROS activity, MAPKs signaling pathway, and subsequent NFATc1 translocation and expression, as well as osteoclast-specific gene expression *in vitro*. *In vivo* study suggested that exposure to GPF prevented osteoporosis-related bone loss in the ovariectomized mice. These findings indicate that GPF attenuates osteoclastogenesis and relieves ovariectomy-induced osteoporosis by inhibiting ROS and MAPKs/c-Fos/NFATc1 signaling pathway. This suggested that GPF may be potentially used to treat bone diseases like periodontitis, rheumatoid arthritis, and osteoporosis associated with osteoclasts.

## Introduction

Physiologically, the maintenance of bone homeostasis remains in a dynamic balance, in which osteoblasts and osteoclasts play a vital role (Harada and Rodan, 2003). This homeostasis balance gets disrupted as a result of diseases such as hyperparathyroidism or Cushing’s disease; as part of natural processes such as menopause or aging; or as a side effect of medications such as corticosteroids. The osteoclast-induced bone resorption outperforms the osteoblast-mediated bone generation, leading to skeletal disorders, such as osteoporosis. Osteoporosis, a common disease affecting a large population worldwide, is characterized by reduced bone mass, destruction of bone microstructure, and increased susceptibility to fragility fracture; it has caused severe morbidity or even mortality to patients, as well as numerous medical and economic burden to our aging population in society (Anne et al., 2001; Harvey et al., 2010; Hernlund et al., 2013).

Osteoclasts, the polynucleated cells generated through the fusion of the mononuclear monocyte/macrophage progenitors, have a unique function of resorbing and degrading the mineralized bone matrix (Boyle et al., 2003). Typically, M-CSF and RANKL are the two critical molecules for promoting osteoclastogenesis. M-CSF mostly activates the c-Fms receptor to facilitate the early mononuclear precursor proliferation and survival (Teitelbaum, 2000). RANKL, by binding to its receptor RANK, activates mature osteoclasts and modulates osteoclast formation from mononuclear precursors in the presence of M-CSF (Lacey et al., 1998; Nakagawa et al., 1998). After the binding of RANKL onto RANK, Tumor-necrosis factor (TNF) receptor-associated factor 6 (TRAF6) is recruited to the RANK cytoplasmic domain, thereby activating the signal transduction cascades such as the NF-κB and the MAPK pathways (Lee et al., 2002; Boyle et al., 2003). The extracellular signal-regulated kinase (ERK) signal transduction pathway plays an important part in regulating the migration, bone resorption, and differentiation of osteoclasts (Nakamura et al., 2003; He et al., 2011). RANKL-induced activated ERK phosphorylates c-Fos at its C-terminal domain on ser362 and ser374 (Wagner and Matsuo, 2003; Amcheslavsky and Bar-Shavit, 2007). RANKL-induced activated JNK induces the expression of two crucial osteoclastogenic transcription factors, namely c-Fos and NFATc1 (Chang et al., 2008; Park et al., 2015). Besides, JNK signaling induces phosphorylation of c-Jun, a transcription factor (TF) that can constitute a complex together with c-Fos (Grigoriadis et al., 1994; Ikeda et al., 2008). JNK-activated c-Jun signaling plays critical roles in the regulation of the RANKL-mediated differentiation of osteoclasts and the nuclear factor of activated T cells (NFAT) family (Ikeda et al., 2004). c-Fos is an important part of the TF complex AP-1, which has been recognized to be essential for significantly inducing NFATc1 for its direct regulation on the transcription of NFATc1 (Takayanagi et al., 2002; Wagner and Eferl, 2005). NFATc1 is recognized as a master TF; its induction and activation integrate the RANKL signal transduction in osteoclast terminal differentiation (Takayanagi et al., 2002). Thus, the RANKL-mediated MAPKs signal transduction pathway activation facilitates activation of NFATc1 and c-Fos, which later modulates osteoclast-specific gene transcription, finally causing mature osteoclast formation (Takayanagi et al., 2002; Boyle et al., 2003; Asagiri et al., 2005).

Increasing evidence demonstrates that ROS plays a vital role in RANKL-induced osteoclastogenesis (Lee et al., 2005; Li et al., 2020; Wang et al., 2020) and enhances osteoclastic bone resorption function *in vitro* and *in vivo* (Garrett et al., 1990; Bax et al., 1992). ROS are likely to act as second messengers in RANKL signaling essential for osteoclastogenesis. During osteoclast differentiation, RANKL stimulation increases intracellular ROS production in BMMs by a signal transduction cascade that involves TRAF6, Nox1, and Rac1. RANKL-induced ROS production, activation of MAPKs pathways, and TRAP-positive osteoclast differentiation were blocked in various degrees in precursors when any part of the TRAF6/Rac1/Nox1 signaling cascade got interrupted, which indicates that ROS are required and crucial for osteoclastogenesis (Lee et al., 2005). This finding corroborates the opinion that MAPKs activation probably can be modulated by ROS, resulting in the alteration of gene expression (Torres, 2003). Furthermore, it is suggested that antioxidants can inhibit the formation of osteoclasts and protect against bone loss induced by estrogen deficiency through boosting the oxidant defenses (Lean et al., 2003; Chen et al., 2019). Thus, attenuating the formation of ROS may significantly inhibit the formation of osteoclasts and effectively improve osteoporosis. Regulating intracellular ROS signaling might be a crucial treatment intervention for osteoporosis.

6′-O-Galloylpaeoniflorin, a compound from peony root-extracted acylated monoterpene glucoside, is constituted by D-glucose, benzoyl, and galloyl moieties (Kang et al., 1991). Nowadays, it is thought that galloyl and benzoyl moieties, which are distributed in some natural catechins in tea, are the major determinants of anticancer, antimicrobial, and antioxidant effects (Silva et al., 2002; Taguri et al., 2006; Rivero-Buceta et al., 2015). Recently, GPF has been shown to possess the antitumor (Zheng et al., 2018; Gao et al., 2020), antiplatelet aggregation (Liao et al., 2018b), anticoagulation (Koo et al., 2010), antiallergic potential (Shi et al., 2016), as well as angiogenic effects (Liao et al., 2018a). Besides, it can reduce the inflammatory response, cellular injury, and apoptosis *in vitro* (Yao et al., 2013; Song et al., 2014; Wen et al., 2018; Lu et al., 2019). Furthermore, GPF has been identified with the radical scavenging and antioxidant effects and can inhibit phenyl hydroquinone-induced oxidative DNA damage (Okubo et al., 2000; Matsuda et al., 2001; Wen et al., 2018). Yao et al. reported that GPF protected against cell damage resulting from oxidative stress (OS) in human keratinocytes (Yao et al., 2013). Also, network pharmacology to unveil the biological basis of GPF in cancer treatment has demonstrated that GPF downregulates gene expression in the MAPK signaling pathway (Zheng et al., 2018). GPF may have an antioxidant effect, but its anti-osteoclastogenic activity and anti-osteoporosis function necessitate further validation.

After considering the influence of ROS on osteoclastogenesis and osteoclast capability of absorbing bone tissue, as well as the antioxidant and radical-scavenging activity of GPF, we hypothesized that GPF might attenuate osteoclastogenesis and have potential effects on osteoporosis via inhibition of intracellular ROS signaling by antioxidant mechanisms.

The current study showed that GPF suppressed the formation of osteoclasts and bone resorption induced by RANKL depending on its dose *in vitro* and protected mice from ovariectomy-induced osteoporosis *in vivo*. GPF exerted antioxidant effects and attenuated RANKL-mediated intracellular ROS production. GPF exposure was found associated with decreased activation of NFATc1 and MAPK pathways induced by RANKL, subsequently leading to inhibition of osteoclast-special gene expression. Overall, our study suggested that GPF has the potential to serve as a novel and promising candidate for treating osteoclast-related bone diseases such as osteoporosis.

## Materials and Methods

### Reagents

Alpha modification of Eagle’s medium (α-MEM) were purchased from HyClone (GE Healthcare Life Sciences, Chicago, IL, United States). Fetal bovine serum (FBS), penicillin, and streptomycin were provided by Gibco (Thermo Fisher Scientific, Waltham, MA, United States). GPF, provided by Chengdu Must Bio-Technology (Chengdu, China), was dissolved in dimethyl sulfoxide. Antibodies specific for ß-Actin, p38, phospho (p)-p38, ERK, p-ERK (1:2,000), JNK, p-JNK, IκBα, p-IκBα, NF-κB p65, p-NF-κB p65, and c-Fos, together with the anti-rabbit or anti-mouse fluorescence-labeled IgG secondary antibodies were provided by Cell Signaling Technology (CST, Danvers, MA, United States). Antibodies specific for NFATc1 (1:250) was provided by Santa Cruz Biotechnology (Dallas, TX, United States). All antibodies were diluted at 1:1,000 unless otherwise indicated. Phenylmethylsulfonyl fluoride, RIPA cell lysis buffer, BCIP/NBT Alkaline Phosphatase Color Development Kit, Cell Counting kit-8, phosphatase inhibitors, and protease inhibitors were provided by Beyotime Institute of Biotechnology (Shanghai, China). The recombinant molecules: RANKL and M-CSF were provided by R&D Systems (Minneapolis, MN, United States).

### Cell Culture

The non-adherent bone marrow-derived macrophages/monocytes were obtained from tibias and femurs in the male C57BL/6J rats (5 weeks old), which were then inoculated into medium A (α-MEM containing 10% FBS, 50 ng/ml M-CSF and 1% PS). After four days of culture, cells were rinsed by PBS twice; then those adherent cells were collected as BMMs for experimental purposes.

### Cell Proliferation and Viability Assays

BMMs (indicated cell density, 8 × 10^3^ cells/well) dependent on M-CSF were inoculated into a 96-well plate and were incubated overnight. After replacing the medium, GPF was added at specific doses. Ninety six hours later, all wells were added with 10 µL CCK-8 solution, and the cells were subjected to 2 h of incubation in the dark under 37°C. Later, the Cytation five Cell Imaging Multi-Mode Reader (BioTek, Vermont, United States) was employed to detect the absorbance (OD) value at 450 nm. Besides, for each sample treated with GPF, the blank OD_450_ value was taken away. Then, the OD_450_ value of each sample treated with GPF was divided by the control value and then multiplied by 100 to determine the viable or proliferating cell percentage. The formula is as follows:

$$\%\text{viable}\;\text{cells}=\left({\text{absorbance}}_{\text{GPF}-\text{treated}\;\text{sample}}-{\text{absorbance}}_{\text{blank}}\right)/\left({\text{absorbance}}_{\text{control}}-{\text{absorbance}}_{\text{blank}}\right)\times 100.$$

### Osteoclastogenesis and Tartrate-Resistant Acid Phosphatase Staining

To generate osteoclasts, BMMs were seeded in 96-well plates at indicated cell density and were cultured in medium B (medium A plus 50 ng/ml RANKL, also called “induced medium”) with indicated concentrations of GPF for seven days. We replaced medium at intervals of two days till matured osteoclasts were formed. Thereafter, cells were subjected to 4% paraformaldehyde (PFA) fixation for 15 min and then stained to detect the enzymatic activity of TRAP by using the TRAP-staining kit. Finally, osteoclasts were determined as cells having at least three nuclei. Images were captured by a Cytation 5 Cell Imaging Multi-Mode Reader.

### Podosome Belt Formation

The same culture conditions (8 × 10^3^ cells/well in 96-well plates, with or without GPF) were applied to osteoclastogenesis. When mature osteoclasts were formed, they were subjected to 15 min of 4% PFA fixation, washing by PBS thrice and 15 min of permeabilization using 0.1% TritonX-100. Thereafter, cells were subjected to 1 h of 0.25% bovine serum albumin (BSA) treatment, followed by 2 h of incubation in the dark using Rhodamine-Phalloidin. Following washing with PBS three times, cells were stained with DAPI for 6 min. Images were captured by fluorescence microscopy.

### Resorption Pit Assay

To observe the bone-resorptive function of osteoclasts *in vitro*, a resorption pit assay was performed. BMMs were then inoculated into 6-well plates, followed by five days of incubation using the induced medium. After the formation of small cells similar to osteoclasts, they were taken out of the 6-well plates, and an equivalent number of cells were inoculated in the 96-well hydroxyapatite plates at the indicated cell density and were cultured in an induced medium with indicated concentrations of GPF for about four days. After fixing 1/2 wells, cells were subjected to staining to detect the TRAP activity. In the remaining 1/2 wells, cells were bleached and removed with hypochlorous acid. Wells were washed thrice with sterile water, air-dried, and then captured using a Cytation 5 Cell Imaging Multi-Mode Reader. Resorbed area per well was measured by Image J (NIH, Bethesda, Maryland, United States) and was used to evaluate the osteoclast function.

### Quantitative Real-Time Reverse Transcription-Polymerase Chain Reaction

BMMs were plated in 6-well plates (2 × 10^5^ cells/well) and incubated in an induced medium with or without GPF. When osteoclasts matured, the RNAiso Plus was used to extract cellular RNA in accordance with specific protocols. Then, 2 µg total RNA was used to prepare cDNA through reverse transcription, with the use of 4 µL of 5 × primescript RT Enzyme MIX in a total volume of 20 µL by supplementing RNase free distilled water. Afterward, the quantitative real-time PCR assay was performed on the qTOWER^3^ Real-time PCR Thermal Cycler by using TB Green Premix Ex Taq. The PCR conditions were as follows: 5 min under 95°C; 10 s under 95°C, 20 s under 60°C, and 20 s under 72°C for 30 cycles; and finally 10 min of elongation under 72°C. To normalize the relative gene expression, GAPDH was used as a reference gene. Primer sequences are listed in Table 1.

**TABLE 1:** Primer sequences used for mRNA transcript analysis.

Gene name	Forward sequence	Reverse sequence
GAPDH	AGG​TCG​GTG​TGA​ACG​GAT​TTG	GGG​GTC​GTT​GAT​GGC​AAC​A
*c*-*Fos*	GCGAGCAACTGAGAAGAC	TTGAAACCCGAGAACATC
NFATc1	CAA​CGC​CCT​GAC​CAC​CGA​TAG	GGC​TGC​CTT​CCG​TCT​CAT​AGT
*Acp5*	TGTGGCCATCTTTATGCT	GTCATTTCTTTGGGGCTT

### Western Blot Analysis

BMMs (5 × 10^5^ cells/well) were inoculated into the 6-well plates for overnight incubation. After 2 h of serum starving, cells were incubated with *a*-MEM with or without GPF for an additional 2 h. Then, RANKL stimulation was performed at specific times (0, 5, 10, 20, 30, and 60 min). After washing with PBS, the RIPA cell lysis buffer that contained PMSF, a phosphatase inhibitor, and protease inhibitor was used to lyse cells. After 15 min of centrifugation of the lysates at 12,000 rpm, the supernatants were harvested for further analysis. SDS-PAGE gels were used to separate the proteins. After migration, the Trans-Blot Turbo Transfer System (Bio-Rad Laboratories, Hercules, CA, United States) was used to transfer the separated proteins onto nitrocellulose membranes. Later, 5% (w/v) skimmed milk powder within TBST that contained 0.05% Tween-20 was used to block the membranes for 2 h under room temperature (RT), followed by overnight incubation using specific antibodies under 4°C. Then, membranes were rinsed by TBST thrice, followed by another 2 h of incubation with fluorescence-labeled secondary antibody. Finally, the ChemiDoc^TM^ MP Imaging System (Bio-Rad Laboratories, Hercules, CA, United States) was used to obtain images.

### Assessment of Intracellular Reactive Oxygen Species

Reactive Oxygen Species Assay Kit was used to measure the intracellular production of ROS. The fluorescence-free DCFH-DA can freely penetrate the cell membrane. It is hydrolyzed by the intracellular esterase after it enters the cells, which generates DCFH that is unable to penetrate the cell membrane. The ROS produced in cells is involved in oxidizing the non-fluorescent DCFH for the production of fluorescent 2′,7′-dichlorofluorescein (DCF). Typically, the DCF fluorescence may be detected to assess the intracellular ROS contents. Upon 100 ng/ml RANKL stimulation with or without GPF at the 10, 20, and 40 µM doses, *a*-MEM that contained 10 µM DCFH-DA was further used to culture BMMs for 1 h in dark. The fluorescence microscope was also used to capture cell images. Then, the Image J software was employed to analyze the average fluorescence intensity in every cell as well as the ROS-positive cell percentage in each field.

To perform flow cytometry, the above procedures were done, and then cells were harvested, centrifuged, and resuspended. Next, we detected the fluorescence intensity at the excitation and emission wavelengths of 488 and 525 nm, respectively, through flow cytometric analysis. A total of 10,000 events were acquired for analysis. Finally, the average value was quantitatively determined.

### Ovariectomy-Induced Osteoporosis Mouse Model

The Institutional Animal Ethics Committee of the Second Affiliated Hospital, Shantou University Medical College, China, approved all the *in vivo* experiments, which conformed to all relevant regulatory standards. The mice were maintained under the pathogen-free controlled environment, and at most, five mice were kept in each cage. All animals were allowed to freely drink water and eat a standard rodent chow diet. After acclimation for one week, eighteen C57BL/6J female mice (19.5 ± 1.4 g, 11 weeks old) were randomized into three groups (*n* = 6 each): sham group, OVX group, and OVX + 10 mg/kg GPF group. According to standard procedures, under anesthesia with 3% chloral hydrate via intraperitoneal injection, surgical bilateral ovariectomy was performed on the mice in the OVX + GPF group and OVX group. Ovaries were exteriorized from mice in the sham group rather than their removal. After surgery, every mouse was injected intraperitoneally with 80,000 units of penicillin to prevent infection. One week after postoperative recovery, intraperitoneal injection of the indicated concentration of GPF was delivered to the OVX + GPF group every two days for six weeks. As a vehicle control, normal saline (NS) was injected into mice in both OVX and sham groups. Six weeks later, all mice were killed through cervical dislocation. 4% formaldehyde was used to fix the excised left femurs for histological and Micro-CT analysis.

### Micro-CT Scanning

The distal region of the left femur was used for micro-computed tomography (micro-CT) analysis. Femurs were resected from mice immediately after their sacrifice and were subjected to 24 h of 4% PFA fixation, followed by scanning by the Skyscan 1272 micro-CT scanner (Bruker micro-CT, Kontich, Belgium). The following scanning parameters were set: source current, 142 μA; source voltage, 70 V; rotation step, 0.20 degree; pixel size, 9 µm; and AI, 0.5 mm filter. A region of interest (ROI) 0.5 mm over the dismal femur growth plate; height, 2 mm was selected to analyze the trabecular bone. Then, some bone-associated parameters—bone volume/tissue volume (BV/TV), trabecular thickness (Tb. Th), trabecular number (Tb. N), connectivity density (Conn. Dn), Bone surface (BS), as well as trabecular separation (Tb. Sp)—were examined.

### Histological Analysis

After micro-CT scanning, the EDTA decalcified solution (pH 7.4) was used to decalcify the left femurs for three weeks, followed by dehydration and paraffin embedding. Then, all specimens were sliced into the 4-µm sections by the microtome for H&E staining to observe the bone microstructure of the femur and TRAP for the visualization of osteoclasts. The Nikon microscope (Nikon Corporation, Minato, Tokyo, Japan) was used to capture images.

### Statistical Analysis

All experiments were repeated at least three times. SPSS 19.0 (IBM, United States) was utilized for statistical analysis. The quantitative data were expressed in the manner of mean ± standard deviation (SD). Student’s t-test was used to determine the difference between two groups, and one-way ANOVA was used to assess the difference of more than two groups. A difference of *p* < 0.05 indicated statistical significance.

## Results

### 6′-O-Galloylpaeoniflorin Inhibits Receptor Activator of NF-κB Ligand-Induced Osteoclastogenesis Without Significant Cytotoxicity in the Cultured Cells

**FIGURE 1:**
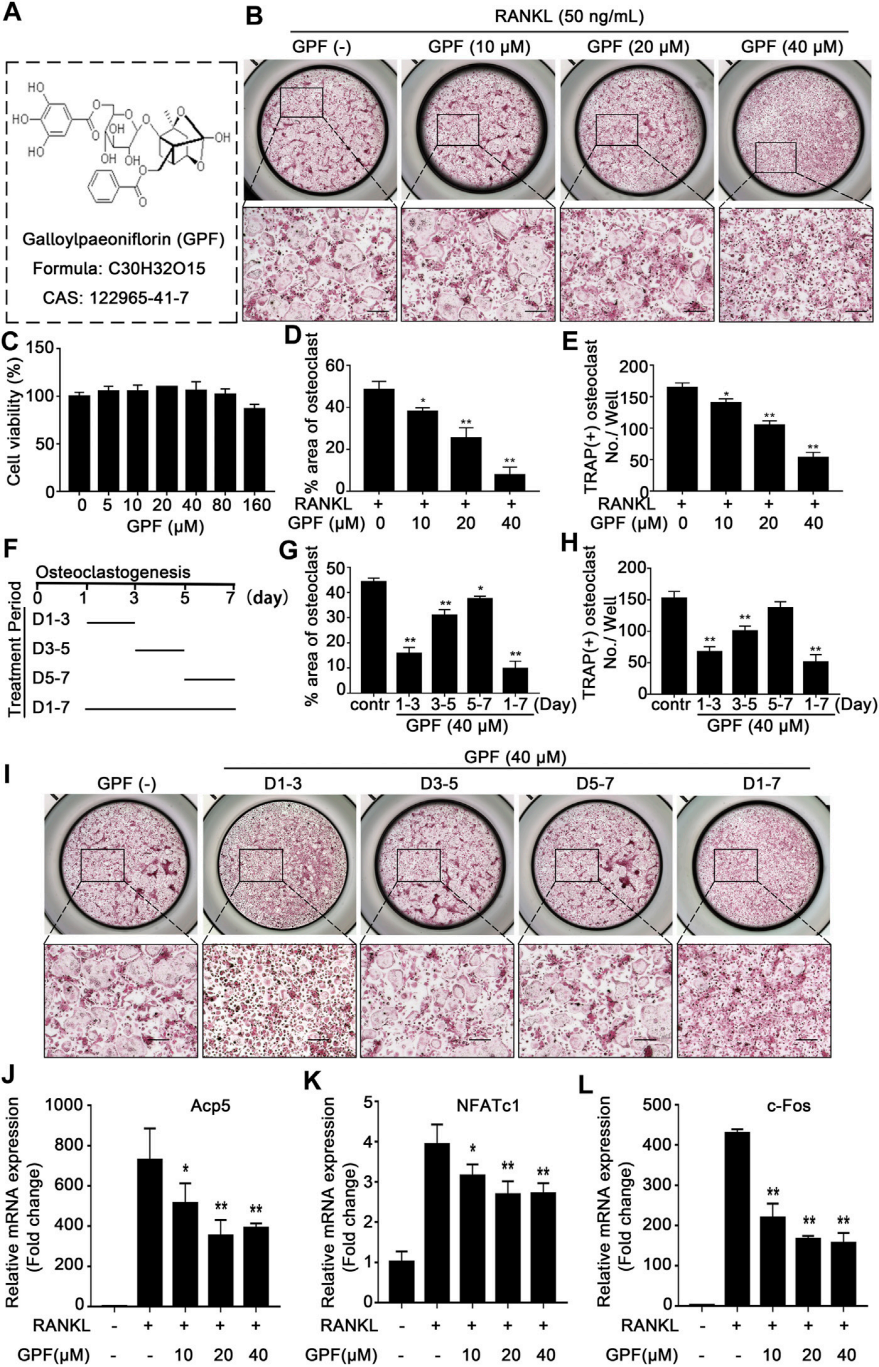
GPF inhibits RANKL-induced osteoclastogenesis *in vitro* without significant cytotoxicity in the cultured cells. **(A)** The molecular structure and CAS number of GPF. **(B)** Representative images showing Inhibitory effects of GPF on osteoclastogenesis in BMMs. BMMs were incubated with RANKL and M-CSF in the absence or presence of GPF (0, 10, 20 and 40 µM). On day 7, cells were fixed and stained for TRAP (*n* = 3). **(C)** CCK-8 assay after treating with varying concentrations of GPF for 96 h (*n* = 3). **(D,E)** Quantitative analysis was performed to detect the total area of TRAP-positive multinucleated cells (nuclei > 3) /well and number of TRAP-positive multinucleated cells/well (*n* = 3). **(F)** The time periods of the treatment of GPF. **(G,H)** Quantification of TRAP-positive multinucleated cells treated with GPF in different time periods. Total area of TRAP-positive cells/well and number of multinucleated osteoclasts were counted (*n* = 3). **(I)** Representative images of TRAP staining showing BMMs treated with GPF for the indicated days during osteoclastogenesis (*n* = 3). **(J-L)** After stimulated with RANKL for 5 days in the presence of 40 µM GPF, osteoclast-specific genes expression of *c*-*Fos*, NFATc1 and *Acp5* relative to GAPDH were quantified by real-time PCR and presented as fold induction. All bar graphs are presented as mean ± SD. *p < 0.05, **p < 0.01 compared with control group (without GPF treatment). One-way ANOVA. Scale bar = 200 µm.

The chemical structure of GPF is demonstrated in Figure 1A. We first tested whether GPF exhibits cytotoxicity against primary BMMs by CCK-8. We found that GPF had an insignificant effect on the viability of BMMs at concentrations below 80 µM (Figure 1C). Therefore, the GPF level was used in later analysis. For assessing how GPF affected the formation of osteoclasts, the medium supplemented with M-CSF and RANKL with or without increasing concentrations of GPF as indicated was used to culture BMMs. Then, TRAP staining suggested that the differentiation of osteoclasts was significantly inhibited (Figures 1B, D,E). 40 µM GPF was added to the culture medium at different stages (early period: day 1–3, middle period: day 3–5, later period: day 5–7, whole period: day 1–7) during the osteoclast differentiation, that facilitated a better understanding of the osteoclast formation degree with the highest sensitivity to GPF exposure (Figure 1F). As a result, inhibition of osteoclastogenesis occurred in all three stages, but more obviously in the early stage (Figures 1G–I).

For better identifying the molecular impact of GPF on osteoclasts *in vitro*, osteoclast-specific gene expressions, including *c-Fos*, NFATc1, and *Acp5*, were tested by quantitative real-time PCR during osteoclast formation with or without GPF treatment. As shown, osteoclast-specific gene expressions of the control group were remarkably upregulated during osteoclast differentiation, but in the GPF group, compared to the control group, these gene expressions were inhibited by GPF in a dose-dependent manner (Figures 1J–L).

### 6′-O-Galloylpaeoniflorin Inhibits Podosome Belt Formation and Bone-Resorptive Function of Osteoclasts *in vitro*

**FIGURE 2:**
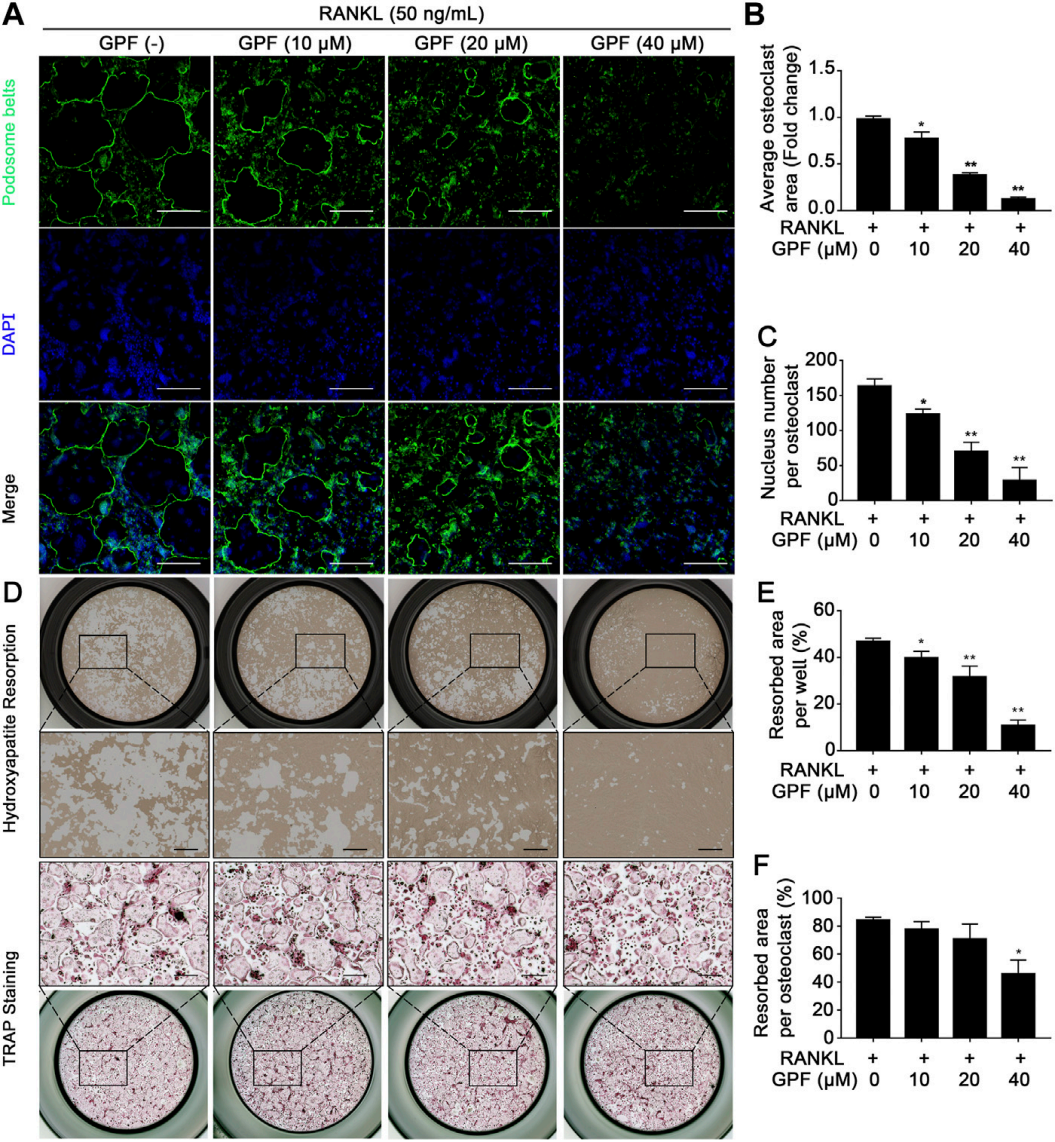
GPF inhibits podosome belt formation and bone-resorptive function of osteoclasts. **(A)** Representative images showing podosome belt formation in osteoclasts treated with GPF. podosome belt (green) and nuclei (blue) staining of osteoclasts. **(B,C)** Quantification of average osteoclast area and the nuclei number per osteoclast (*n* = 3). **(D)** Representative images showing the osteoclastogenesis and hydroxyapatite resorption. Pre-osteoclast-like cells were seeded in hydroxyapatite-coated plates and treated by RANKL with or without GPF. After fixing 1/2 wells, cells were subjected to staining to detect the TRAP activity. In the remaining 1/2 wells, cells were bleached and removed with hypochlorous acid. **(E,F)** Quantification analyses of resorbed area per well and resorbed area per osteoclast (*n* = 3). All bar graphs are presented as mean ± SD. *p < 0.05, **p < 0.01 compared with control group (without GPF treatment). One-way ANOVA. Scale bar = 200 µm.

Rhodamine-phalloidin was used to stain cells for the visualization of morphological alterations and the formation of podosome belts within cells subjected to GPF treatment or not. In line with the suppressed osteoclast differentiation, as mentioned above, the GPF treatment group showed markedly decreased podosome belt area in each field and showed reduced nucleus number in each osteoclast (Figures 2A–C).

We next performed a resorption pit assay to observe the bone-resorptive function of osteoclasts *in vitro*. The osteoclast number and the resorbed area per osteoclast decreased significantly with GPF application (Figures 2D–F). Therefore, the compound exhibited an inhibitory effect on osteoclast bone-resorptive function as the bone resorption assay showed, and possibly inhibited the terminal process when pre-osteoclast-like cells turned into mature osteoclasts. The above findings indicate that GPF suppressed osteoclastogenesis and osteoclast activity.

### 6′-O-Galloylpaeoniflorin Scavenges Receptor Activator of NF-κB Ligand-Mediated Intracellular Reactive Oxygen Species Generation

**FIGURE 3:**
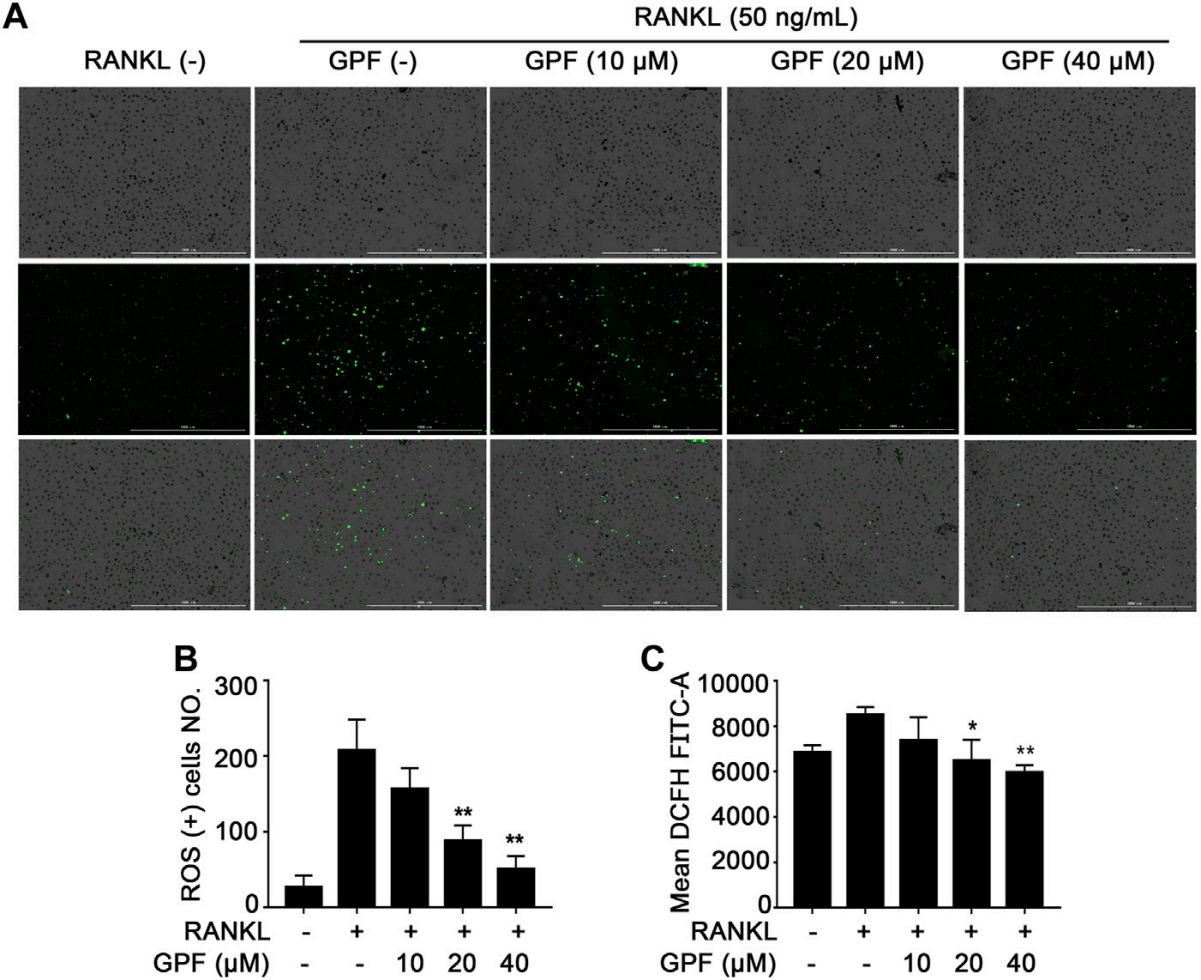
GPF attenuates RANKL-induced ROS generation *in vitro*. **(A)** Representative images of ROS production induced by RANKL in BMMs when pre-treated with or without GPF. **(B)** The quantification of ROS production was calculated (*n* = 3). **(C)** Mean DCFH FITC-A induced by RANKL in BMMs when pre-treated with or without GPF. All bar graphs are presented as mean ± SD. *p < 0.05, **p < 0.01 compared with control group (without GPF treatment). One-way ANOVA. Scale bar = 1000 µm.

To examine the interfering effect of GPF on the RANKL-mediated ROS generation in cells during osteoclast differentiation, a Reactive Oxygen Species Assay kit was used. As cell images showed, stimulation of BMMs increased the intensity of DCF fluorescence. GPF dose-dependently inhibited RANKL-stimulated ROS production in BMMs (Figures 3A,B). Furthermore, flow cytometry analysis also indicated that treatment with GPF inhibited the RANKL-mediated increase in intracellular ROS (Figure 3C). Thus, current findings suggested that GPF suppressed osteoclastogenesis probably by scavenging intracellular RANKL-generated ROS in osteoclast precursors.

### 6′-O-Galloylpaeoniflorin Suppresses Receptor Activator of NF-κB Ligand-Induced Activation of c-Jun N-Terminal Kinase and Extracellular Signal-Regulated Kinase Pathways

**FIGURE 4:**
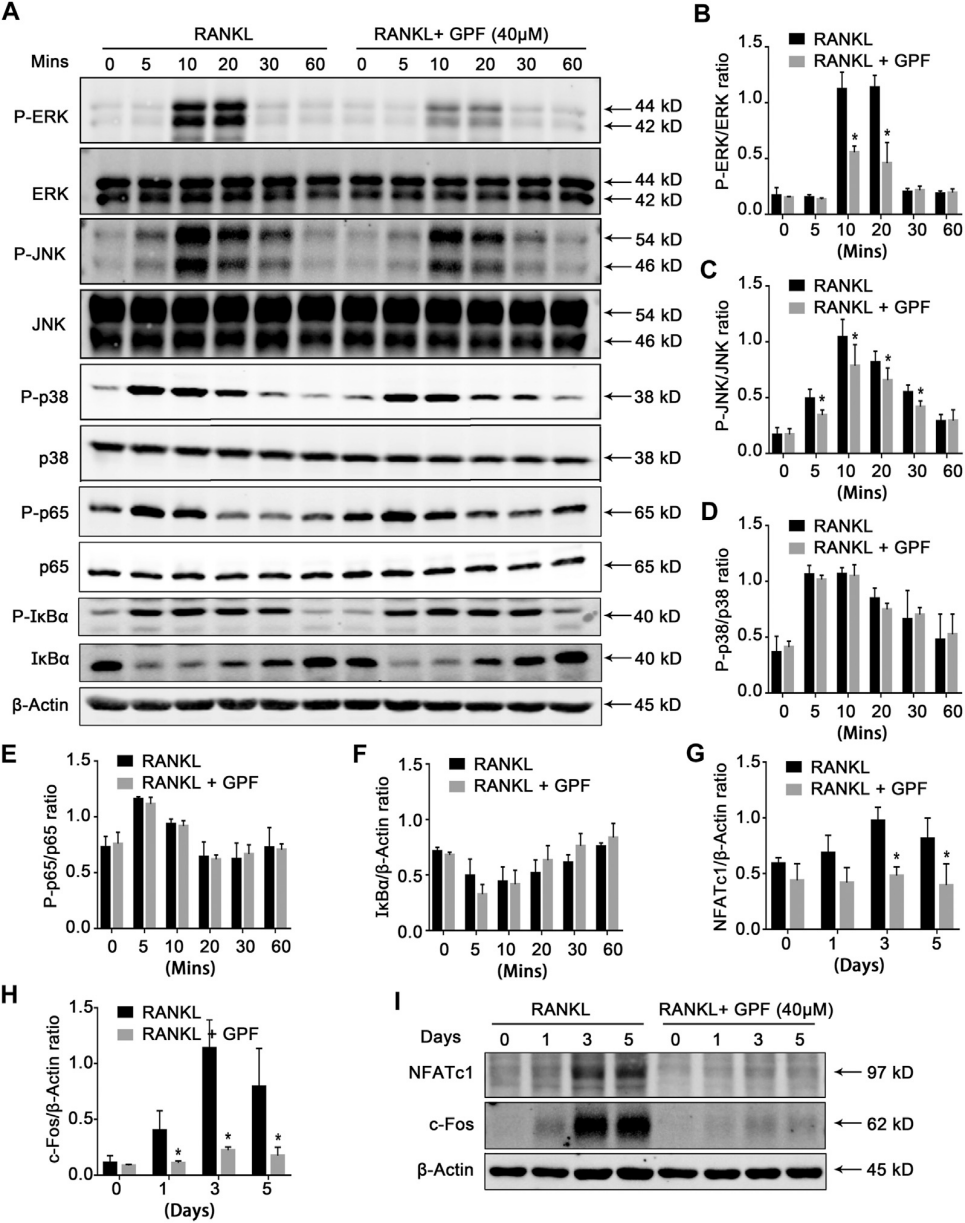
GPF inhibits MAPK, c-Fos and NFATc1 signaling pathways. **(A)** Representative Western Blot images of the effects of GPF on RANKL-induced activation of MAPK, c-Fos and NFATc1 signaling pathway. BMMs were pretreated with 40 μM GPF for 2 h prior to the addition of RANKL at the indicated time points and the indicated proteins were determined. **(B–D)** Quantitative analyses of P-ERK, P-JNK and P-p38 were normalized to total ERK, JNK and p38 respectively (*n* = 3). **(E,F)** Quantification of the ratios of band intensity of IκBα and P-p65 relative to ß-actin (*n* = 3). **(G,H)** Quantification of the ratios of band intensity of NFATc1 and c-Fos relative to ß-actin (*n* = 3). **(I)** BMMs were stimulated by RANKL with or without 40 µM GPF for 0, 1, 3, and 5 days for Western Blot. Representative Western Blot images of the expression levels of NFATc1 and c-Fos. All bar graphs are presented as mean ± SD. *p < 0.05, **p < 0.01 compared with the control group (without GPF treatment). All proteins were compared by Student’s t-test.

This study determined how GPF affected the MAPK as well as NF-κB pathways to better illustrate the mechanism underlying inhibition of the GPF on osteoclastogenesis. With regard to the MAPK pathways, RANKL down-regulation increased the phosphorylation levels of JNK, p38, and ERK (Figure 4A). It was observed that GPF inhibited phosphorylated ERK at 10–20 min (Figure 4B) and JNK at 5–30 min (Figure 4C) but had little effect on phosphorylated p38 (Figure 4D). We also found that GPF made no difference to RANKL-activated p65 and IκBα (Figures 4A,E,F). Collectively, our data suggested that GPF influence osteoclastogenesis probably by inhibiting RANKL-induced activation of ERK and JNK, rather than p38 and NF-κB pathways.

### 6′-O-Galloylpaeoniflorin Inhibits Receptor Activator of NF-κB Ligand-Mediated Activation of c-Fos and NFATc1

We next examined whether GPF regulates activation of c-Fos and NFATc1. According to Figures 4G–I, RANKL-induced NFATc1 and c-Fos protein expression levels were significantly suppressed by GPF. Overall, the findings in this study suggested that GPF might scavenge the RANKL-triggered ROS production and attenuate the RANKL-regulated JNK and ERK signaling activation to suppress the activation of the downstream c-Fos and NFATc1. Thus, this affected osteoclast-specific gene expression, finally suppressing osteoclast formation and bone resorption activity.

### 6′-O-Galloylpaeoniflorin Relieves Ovariectomy-Induced Osteoporosis

**FIGURE 5:**
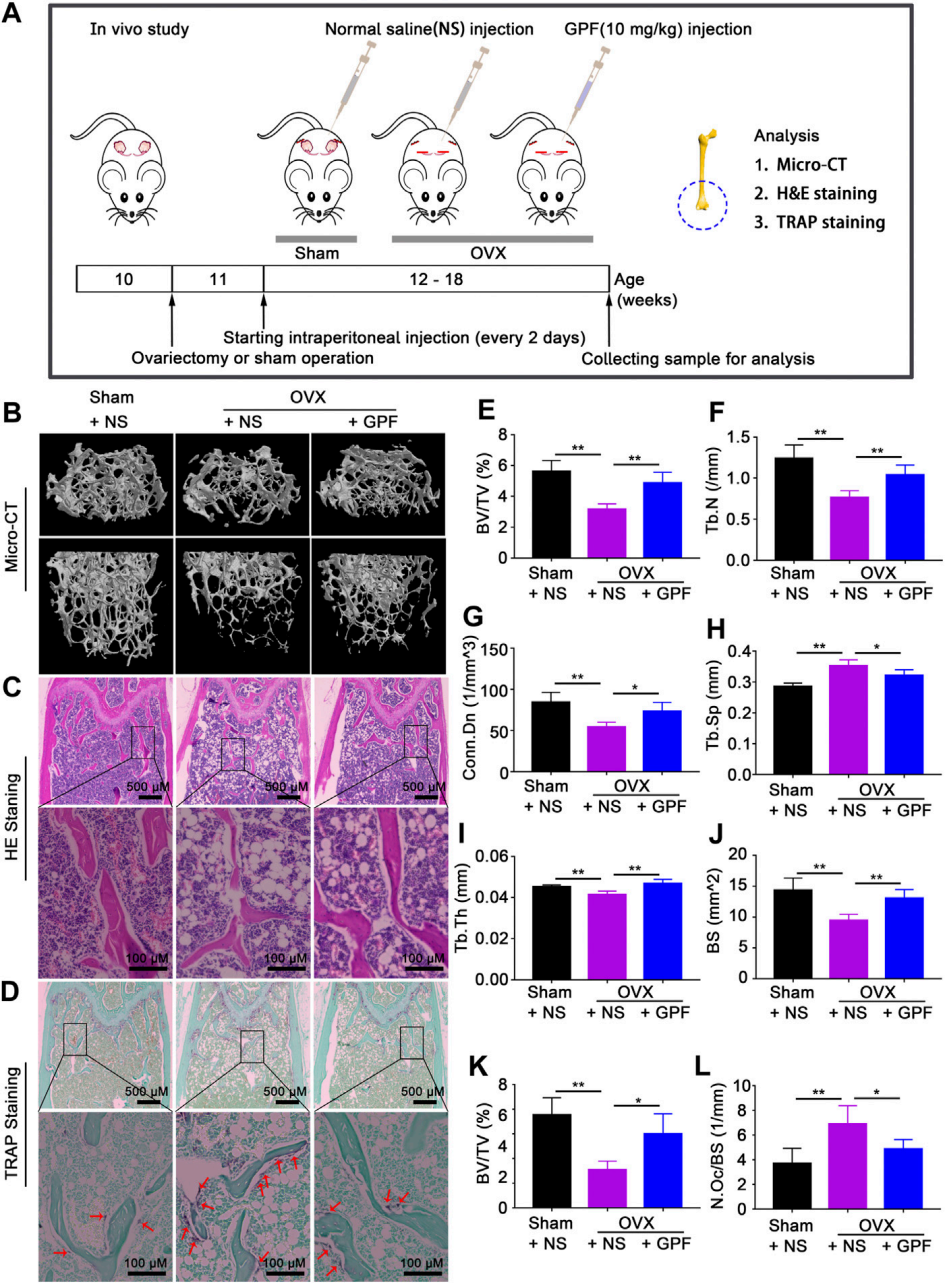
GPF relieves ovariectomy-induced osteoporosis *in vivo*. **(A)** Protocol of the *in vivo* experiment and sensitive techniques for evaluating therapeutic efficacy of GPF. **(B)** Representative micro-CT images of femurs exerting that GPF treatment prevents OVX-induced bone loss. **(C,D)** Representative images of H&E, TRAP staining of decalcified bone sections. **(E–J)** Quantitative analyses of parameters regarding bone microstructure, including BV/TV, Tb.N, Conn. Dn, Tb. Sp, Tb.Th, and BS (*n* = 6). **(K,L)** Quantitative analyses of all bone sections, including BV/TV and N. Oc/BS (*n* = 6). All bar graphs are presented as mean ± SD. *p < 0.05 and **p < 0.01 between the indicated groups. One-way ANOVA.

Having demonstrated that GPF inhibits osteoclast formation and bone resorption, we hypothesized that GPF might ameliorate ovariectomy-induced osteoporosis. As shown in Figure 5A, the mice underwent OVX- or sham-surgery and were given an intraperitoneal injection of 10 mg/kg GPF or normal saline (NS) every two days for six weeks after postoperative recovery. GPF injections relieve ovariectomy-induced bone loss as demonstrated by micro-CT analysis (Figure 5B). Relevant bone parameters were measured, and quantitative analysis showed the increased BV/TV, BS, Tb. N, Tb. Th and Conn. Dn levels, whereas decreased Tb. Sp level of OVX + GPF group in comparison with the OVX + NS group (Figures 5E–J). Also, GPF could obviously relieve ovariectomy-induced bone loss, as was exhibited by HE staining (Figures 5C, K). Then, TRAP staining (Figure 5D) was done to investigate the role of GPF on osteoclast formation during osteoporosis. The mean TRAP-positive cell percentage per bone surface were respectively measured. It showed that, when compared with the NS-treatment group, GPF treatment significantly decreased osteoclastogenesis (Figure 5L).

## Discussion

Currently, osteoporosis treatment strategy includes the reduction of bone resorption and the promotion of bone formation. Principal classes of anti-osteoporosis medications, such as bisphosphonates, estrogens, selective estrogen receptor modulators, and monoclonal antibodies, have shown anti-fracture efficacy (Chen and Sambrook, 2011). However, these drugs are clinically proven to have some side effects (Chen and Sambrook, 2011; Pazianas and Abrahamsen, 2016; Lim and Bolster, 2017; Datta-Mannan, 2019). Therefore, it is still crucial to explore novel medications for osteoporosis treatment. Recent studies revealed the important role of ROS in the RANKL-mediated differentiation of osteoclasts (Chen et al., 2019; Liu et al., 2019). Previous studies demonstrated that GPF exerts antioxidant and radical-scavenging activity (Okubo et al., 2000; Matsuda et al., 2001; Wen et al., 2018). Thus, we hypothesized that GPF might attenuate osteoclastogenesis and have potential effects on osteoporosis by inhibiting ROS and explored the potential mechanisms. The current study showed that GPF attenuates osteoclastogenesis and prevents ovariectomy-induced osteoporosis via inhibiting ROS and MAPK/c-Fos/NFATc1 signaling pathway.

Key findings in the current study:

(1) GPF suppresses the RANKL-mediated formation of osteoclasts depending on its dose, especially at the early stage, with no significant cytotoxicity;
(2) GPF attenuates bone-resorptive function of mature osteoclasts *in vitro*;
(3) GPF suppresses osteoclast-specific gene expression;
(4) GPF markedly inhibits the activation of ERK, JNK, c-Fos, and NFATc1;
(5) GPF scavenges RANKL-mediated intracellular ROS generation; and
(6) GPF relieves ovariectomy-induced osteoporosis.

Firstly, cell viability was analyzed by CCK-8 and intriguingly, with increasing GPF level, the cell viability first increased and then decreased. Such phenomenon was possibly related to the dose-response hormesis, where the low dose had stimulating effect, whereas the high dose had inhibiting effect (Calabrese et al., 2010; Calabrese et al., 2020). As suggested by the RANKL-mediated formation of osteoclasts, GPF markedly suppressed osteoclastogenesis depending on its dose. Then podosome belt formation and Resorption pit assay exhibited that GPF possessed the potential capacity of suppressing osteoclastic resorption. Resultantly, GPF exerts significant potential effects on osteoporosis by its remarkable inhibition of osteoclast formation and function. To further unravel underlying mechanisms, intracellular ROS level, NF-κB pathway, MAPK pathway, and NFATc1 pathway, as well as the expression of the osteoclast-specific gene, were investigated in the current study.

Recently, the evidence has started mounting that RANKL-activated intracellular ROS probably acts as an upstream component of signaling pathways that regulate the process of osteoclastogenesis (Ha et al., 2004; Lee et al., 2005). Scavenging ROS were found to effectively restrain osteoclastogenesis and bone resorption (Chen et al., 2019). Previous studies showed that GPF had antioxidant and radical-scavenging activity (Okubo et al., 2000; Matsuda et al., 2001; Yao et al., 2013; Wen et al., 2018). Therefore, assessments of intracellular ROS were performed. Our results demonstrated that GPF inhibited the RANKL-induced intracellular ROS in a dose-dependent manner; this indicated that GPF may scavenge the RANKL-mediated ROS production in cells to suppress osteoclastogenesis as well as bone resorption. The ROS downstream targets within the RANKL-mediated signal transduction pathway remain uninvestigated, but recent studies indicate that the high ROS levels promote osteoclast formation and function probably by activating NF-kB and MAPKs signaling pathways (Hyeon et al., 2013; Kim et al., 2017). Thus, GPF may inhibit the formation of osteoclasts as well as bone resorption through regulating the ROS-MAPK signal transduction pathway to some extent.

Binding of RANKL to RANK triggers complex signaling cascades, such as the NF-κB pathway, the MAPK pathway, and NFATc1 pathway, that regulate and control commitment and activation of osteoclasts. The NF-κB pathway has been proven to play a key role in osteoclast formation. In our study, strong NF-κB activation was stimulated by RANKL, in line with the previous study. However, GPF exhibited little effect on the phosphorylation of p65 and IκBα, indicating that GPF probably inhibits osteoclastogenesis by other signaling pathways rather than the NF-κB pathway.

**FIGURE 6:**
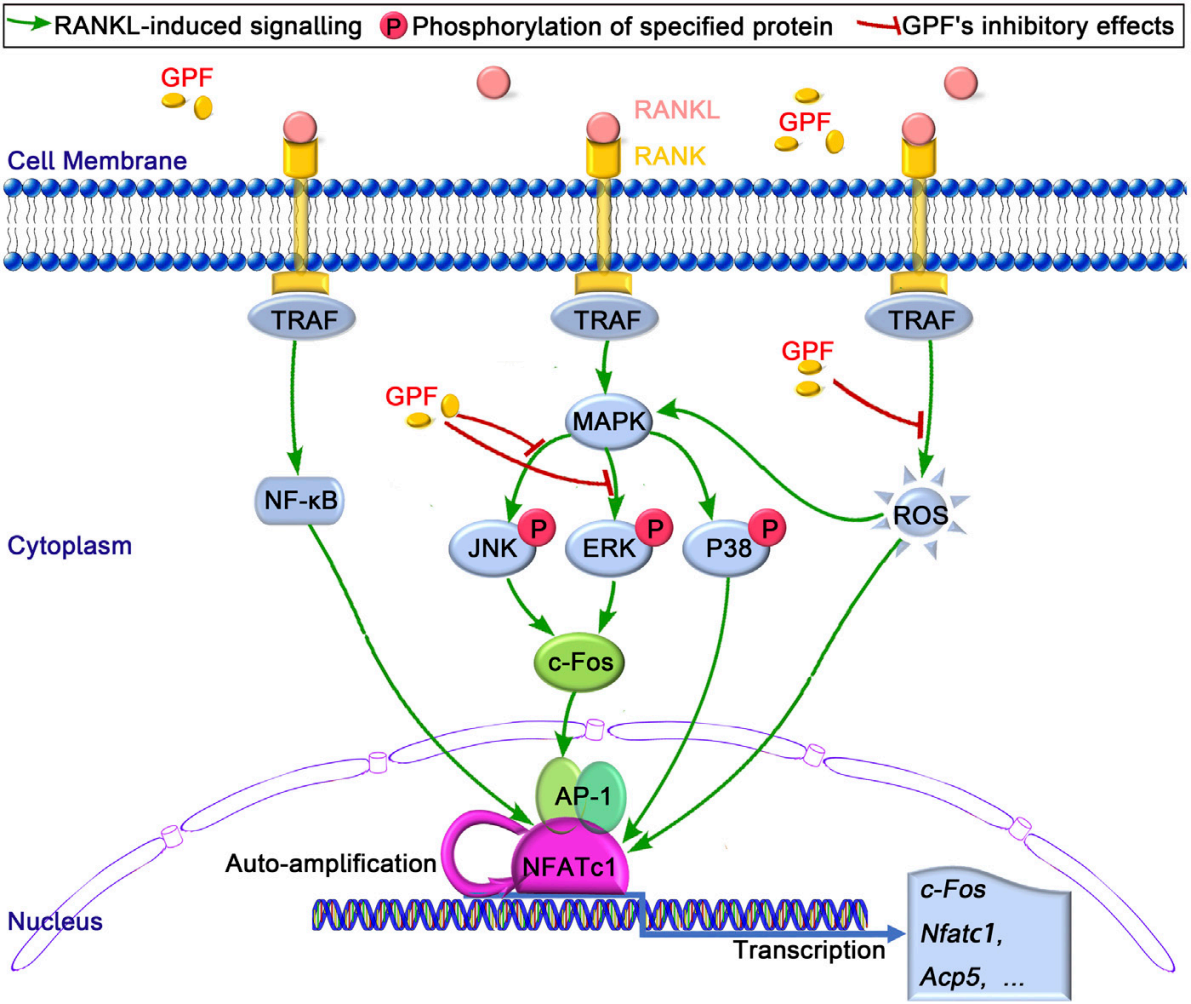
The proposed mechanism by which GPF inhibits osteoclastogenesis. Binding of RANKL to its receptor RANK, results in the recruitment of TRAF, following by the activation of MAPK and NF-κB pathways and the subsequent self-amplification of NFATc1. As a result, several osteoclast-specific genes, including *c-Fos*, *Acp5*, and NFATc1 are upregulated. These signaling events are regulated by RANKL-activated ROS signaling. Our results indicated that GPF can inhibit osteoclast formation and bone resorption via suppressing MAPK (ERK and JNK)/c-Fos/NFATc1 signaling pathways and scavenging ROS activities.

The RANKL-mediated MAPK signal transduction pathway participates in regulating the differentiation of osteoclasts to enable binding to specific DNA response elements and nuclear translocation; thus, achieving the cooperative activation of target gene transcription. The deletion or suppression of such signal transduction cascades is suggested to affect osteoclast formation and function of osteoclast (Ma et al., 2015; Jiao et al., 2017; Kim et al., 2018). Our results showed that GPF attenuated the RANKL-activated ERK and JNK signaling pathway but had little effect on p38 signaling pathways. Both ERK and JNK are crucial pathways during osteoclastogenesis (Lacey et al., 1998; David et al., 2002; Ikeda et al., 2008). RANKL-induced activated ERK phosphorylates c-Fos; thus, mediating the differentiation and function of osteoclast (Wagner and Matsuo, 2003; Amcheslavsky and Bar-Shavit, 2007). Phosphorylation of JNK subsequently activates the transcription factor c-Jun. C-Jun, together with c-Fos, a vital transcription factor during osteoclastogenesis, can form the TF complex, activator protein-1 (AP-1). AP-1 binds to the NFATc1 promoter and regulates its expression. NFATc1, as a master regulator of the osteoclast transcriptome and known for its self-amplification to maintain robust expression, exerts a vital part in the formation and activity of osteoclasts (Takayanagi et al., 2002; Boyle et al., 2003; Asagiri et al., 2005). Findings in several studies indicate that NFATc1 regulates different osteoclast-specific gene expression, such as Cathepsin K(*Ctsk*), *c-Fos*, NFATc1, and *Acp5* (Takayanagi et al., 2002; Kim et al., 2008; Pang et al., 2019). We observed that GPF suppresses the activation of c-Fos and NFATc1. And our qRT-PCR results showed that several osteoclast special genes, including *c-Fos, Acp5*, and NFATc1, were suppressed by GPF. All the above results suggest that GPF inhibits the activation of c-fos via attenuating the phosphorylation of ERK and JNK, which influences the formation of AP-1, the gene-transcriptional regulating function of NFATc1, as well as osteoclast-specific gene levels, thereby suppressing osteoclast formation and activity (Figure 6).

According to the robust *in vitro* findings, we created an ovariectomy-induced osteoporosis mouse model to assess the potential therapeutic effects of GPF *in vivo*.

GPF increased BV/TV, BS, Tb. N, Tb. Th and Conn. Dn values, but decreased Tb. Sp level. Besides, ovariectomy-induced TRAP-positive osteoclasts showed a marked decrease in the bones of OVX + GPF group, compared to OVX + NS group. Based on the results of micro-CT, H&E, and TRAP staining, we highlight that GPF has potential therapeutic efficacy for bone loss in OVX mice. No extra bone tissue for osteoclast-specific gene and protein expression assessment, as well as *in vivo* ROS fluorescence detection, could be collected.

## Conclusion

To sum up, the current study data demonstrated that GPF suppresses the formation of osteoclasts and activity through decreasing the ROS production and suppressing ERK, JNK, c-Fos and NFATc1 signal transduction pathways, followed by the attenuation of downstream osteoclast gene expressions. Moreover, GPF exhibits a noteworthy protective effect on ovariectomy-induced osteoporosis in a mouse model. Additionally, as an effective component of peony root, a crude drug taken in a long time, GPF might be a promising candidate for treatment. These findings provide ample evidence that GPF may serve as a candidate therapeutic to prevent and treat post-menopausal osteoporosis.

## Data Availability

The original contributions presented in the study are included in the article/Supplementary Material, further inquiries can be directed to the corresponding authors.

## Abbreviations

BMMs, bone marrow-derived macrophages/monocytes; cDNA, complementary DNA; FBS, fetal bovine serum; GPF, 6′-O-galloylpaeoniflorin; JNK, c-Jun N-terminal kinase; M-CSF, macrophage colony-stimulating factor; MAPK, mitogen-associated protein kinases; NF-κB, nuclear factor-κ-gene binding; NFATc1, nuclear factor of activated T cell cytoplasmic 1; qRT-PCR, quantitative real-time PCR; RANK, receptor activator of nuclear factor κB; RANKL, receptor activator of nuclear factor κB ligand; ROS, reactive oxygen species; TRAF6, tumor-necrosis factor (TNF) receptor-associated factor 6; TRAP, tartrate-resistant acid phosphatase.
